# Culture filtrate proteome analysis of aminoglycoside resistant clinical isolates of *Mycobacterium tuberculosis*

**DOI:** 10.1186/1471-2334-14-S3-P60

**Published:** 2014-05-27

**Authors:** Divakar Sharma, Hari Shankar, Manju Lata, Beenu Joshi, Krishnamurthy Venkatesan, Deepa Bisht

**Affiliations:** 1 Department of Biochemistry, National JALMA Institute for Leprosy and Other Mycobacterial Diseases (ICMR), Tajganj, Agra, India; 2 Department of Immunology, National JALMA Institute for Leprosy and Other Mycobacterial Diseases (ICMR), Tajganj, Agra - 282004, India

## Background

Aminoglycosides are commonly used in tuberculosis treatment and are drugs of choice especially for MDR patients. They inhibit protein synthesis in susceptible bacteria by interacting with steps of translation. Several explanations have been put forward to explain the mechanism of aminoglycosides resistance but still our knowledge is fragmentary. Many culture filtrate proteins of pathogen represent potential targets for drugs, diagnostic probes and vaccine components; analysis of mycobacterial culture filtrate proteome in relation to aminoglycoside drug resistance is urgently required.

## Methods

*M. tuberculosis* sensitive and resistant clinical isolates were cultured in Sauton’s medium. After four weeks, cells were removed by centrifugation; supernatant was filtered by 0.45 & 0.22µ filters and precipitated using SDS –TCA precipitation method. Culture filtrate proteins were resolved by two dimensional gel electrophoresis and differentially expressed proteins (more than two fold) were selected for identification by matrix assisted LASER desorption/ionization time of flight mass spectrometry (MALDI TOF/MS) and characterization by bioinformatic tools (drug-protein docking).

## Results

On comparing the protein profile of isolates, twelve proteins were found differentially expressed which were identified by MALDI TOF/MS. These proteins were not only involved in various metabolic pathways (site for drug targets) but also involved in survival of mycobacteria (various heat shock proteins).

## Conclusion

These results might help in new drug development through provision of new drug targets and in further revision of therapeutics & vaccines against aminoglycoside drug resistant tuberculosis.

